# Repair of coronary sinus shear stress injury following blunt chest trauma using adenosine-induced asystole

**DOI:** 10.1093/icvts/ivac114

**Published:** 2022-05-04

**Authors:** Rakan I Nazer, Bushr A Mrad, Waseem M Hajjar, Ali M Albarrati

**Affiliations:** 1 Department of Cardiac Science, Unit of Cardiac Surgery, College of Medicine, King Saud University, Riyadh, Kingdom of Saudi Arabia; 2 Department of Surgery, Unit of Trauma, College of Medicine, King Saud University, Riyadh, Kingdom of Saudi Arabia; 3 Department of Surgery, Unit of Thoracic Surgery, College of Medicine, King Saud University, Riyadh, Kingdom of Saudi Arabia; 4 Department of Rehabilitation Science, College of Applied Medical Science, King Saud University, Riyadh, Kingdom of Saudi Arabia

**Keywords:** Blunt chest trauma, Coronary sinus injury

## Abstract

Blunt chest trauma following a motor vehicle accident is the leading cause of non-penetrating cardiac injuries. Major structural heart injuries are fatal due to acute tamponade. We present the case of a 17-year-old male who was involved in a motor vehicle accident. He had an isolated coronary sinus rupture, which was successfully repaired. We propose a potential mechanism implicated in this rare injury, and we summarize a novel repair technique with adenosine-induced transient asystole.

## Introduction

Structural cardiac injuries following blunt chest trauma are associated with high mortality. The ensuing acute tamponade results in death at the scene or in transit to the hospital. An isolated coronary sinus (CS) injury after blunt trauma is rare [1, 2]. Survival is possible with prompt diagnosis and intervention. Repairing an actively bleeding CS can be challenging. In many situations, it requires anticoagulation prior to mechanical circulatory support. Here, we describe an isolated CS rupture, following a blunt trauma, that was successfully repaired without anticoagulation or cardiopulmonary bypass.

## Case presentation

The emergency medical services rushed a 17-year-old male to the emergency department from a motor vehicle accident, where he was ejected from a rolling vehicle. He was found unconscious at the scene. He experienced 4 cardiac arrests, 2 during transport and 2 after arriving at the hospital emergency department; each arrest lasted no more than 2 to 3 min. On arrival, the patient was resuscitated by the trauma team to a state of spontaneous circulation. His Glasgow Coma Scale score was 3/15. His haemodynamic status fluctuated: blood pressure was 60/30 mmHg, heart rate was 130 beats/min. A focused assessment with sonography for trauma showed pericardial and left pleural effusions. Computed tomography (CT) was performed to scan the traumatized areas, but it was abruptly halted due to cardiac arrest. After successfully regaining the pulse with cardiopulmonary resuscitation, the patient was taken immediately to the operating room.

Intraoperatively, an emergency left anterior thoracotomy was performed; it was subsequently extended to a median laparotomy. The pericardium was full of blood. Upon opening the pericardium, the pulse was regained after a short period of manual message. Dark blood emerged from behind the heart. A ruptured CS was identified. Bleeding was exacerbated each time the heart was lifted for visual access. In the abdomen, a retroperitoneal haematoma was identified. The CS bleeding was temporarily controlled by applying pressure.

Next, the team considered administering full anticoagulation with mechanical circulation support to unload the heart. After deliberating, the team determined that anticoagulation carried too high a risk of exacerbating the retroperitoneal bleeding. Instead, the CS bleeding was controlled with intermittent, adenosine-induced transient asystole (12 mg). Asystole facilitated our ability to pass 3–0 polypropylene mattress sutures. Patches of autologous pericardium were used as pledgets. In total, 3 adenosine boluses were required. With each bolus, asystole lasted 2 to 3 s. External cardiac pacing leads were made available for backup pacing. Care was taken to avoid complete occlusion of the CS lumen and injury to the terminal branches of the circumflex coronary artery. After successful repair, the mediastinum and abdomen were packed with sponges and temporarily closed. After 48 h, the patient’s general condition started to improve. On re-exploration there was no expansion of the retroperitoneal haematoma. The chest and abdomen were then closed.

The patient spent 20 days in intensive care. He required a tracheostomy that was later removed. He spent another 40 days in the hospital for extensive rehabilitation. Echocardiograms showed normal biventricular function and no evidence of pericardial effusion. The patient was discharged home in stable condition. Follow-up was uneventful.

## Discussion

**Figure 1:**
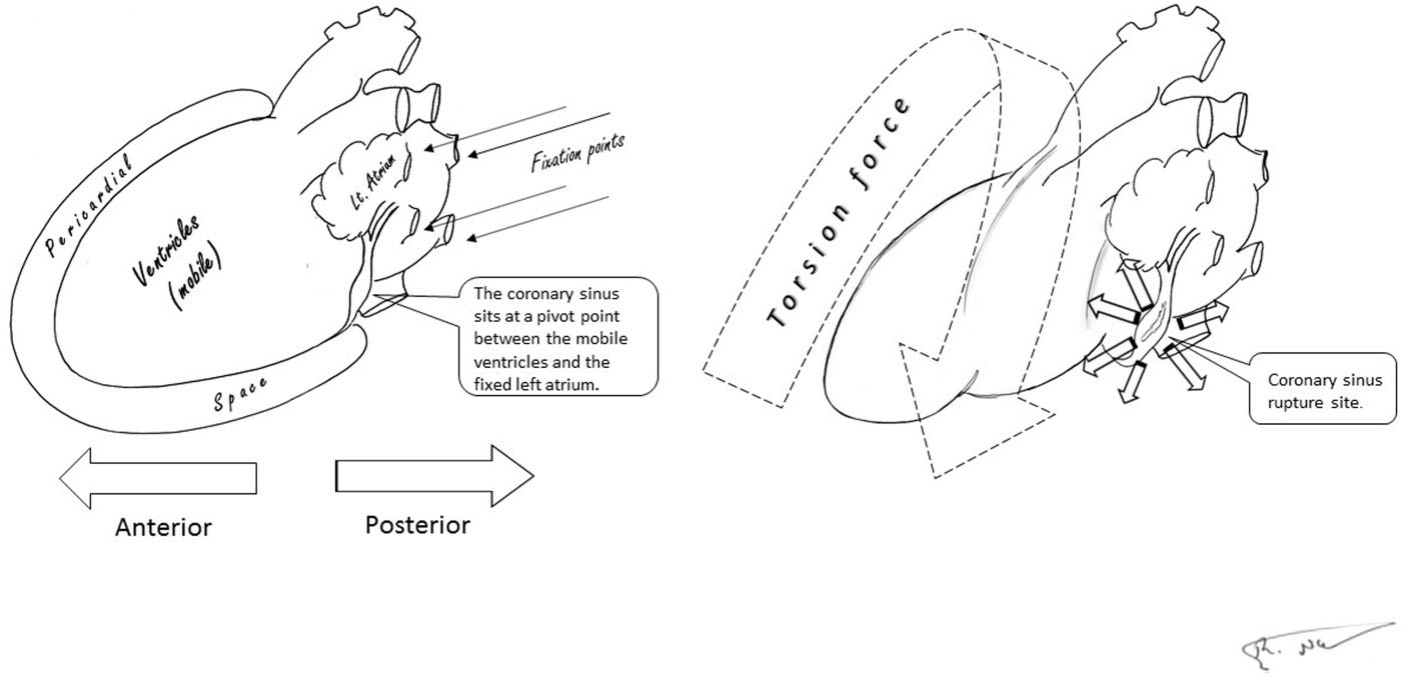
Acceleration-deceleration forces on the heart can lead to torsion stress. The diagram shows (left) the anteriorly situated, mobile ventricular chambers and the left atrium, which is posteriorly fixed (arrows). The coronary sinus (text box pointer) is located at the pivot point between the fixed and the mobile parts (right). In a deceleration injury, the coronary sinus is subjected to torsion force, which can lead to high shear stress and rupture.

Multiple mechanisms have been associated with cardiac injury in blunt trauma. These mechanisms include the direct transfer of mechanical energy from the chest wall to the heart, compression of the chest while the patient is holding his breath and shearing forces that act on the heart, due to a deceleration injury. However, an isolated CS injury is rare. We suggest that this injury might be caused by forces that lead to torsional stress (Fig. 1). Under torsion, the CS sustains high shear stress, which can lead to injury and rupture.

Previous reports that described isolated CS bleeding after a blunt chest injury have advocated supporting the systemic circulation with ECMO and/or a cardiopulmonary bypass. Thus, an arrested, empty heart ensures meticulous haemostasis and CS patency [1, 2]. This condition requires a cardiac surgery set-up, which might not be available in all emergency departments/trauma centres. It also requires anticoagulation with heparin prior to initiating mechanical circulatory support, which can exacerbate bleeding from other injured sites. Adenosine-induced asystole is one option to induce transient asystole to facilitate the repair of a ruptured CS, and it can be safely reproduced in any operating room.

Isolated CS ruptures can result from deceleration injuries due to shear stress. We described a novel technique that provided safe access to the CS during a transient adenosine-induced arrest. This approach might be considered where the bleeding risk is high or mechanical circulatory support is not available. 

**Funding:** No funding was required to support this study.

Conflict of interest: None declared. 

**Consent**: Written informed consent was obtained from the patient and his guardian.

## References

[1] Kim DW , Lee KS, Na KJ, Oh SG, Jung YH, Jeong IS. Traumatic rupture of the coronary sinus following blunt chest trauma: a case report. Journal of cardiothoracic surgery. 2014;9:164. 25409742 10.1186/s13019-014-0164-y PMC4246540

[2] Kim TY , Kim KH. Successful repair of coronary sinus rupture presenting as cardiac tamponade following blunt chest trauma. Interactive cardiovascular and thoracic surgery. 2019;28(6):999–1000. 30508174 10.1093/icvts/ivy323

